# The repeated bout effect of traditional resistance training on cycling efficiency and performance

**DOI:** 10.1007/s00421-024-05422-5

**Published:** 2024-02-20

**Authors:** Baily Devantier-Thomas, Glen B. Deakin, Fiona Crowther, Moritz Schumann, Kenji Doma

**Affiliations:** 1 James Cook Drive, Rehab Sciences Building (DB-43), James Cook University, Townsville, QLD 4811 Australia; 2 Department of Sports Medicine and Exercise Therapy, Chemnitz University of Technology, Chemnitz, Germany

**Keywords:** Exercise-induced muscle damage, Cycling performance, Efficiency

## Abstract

**Purpose:**

This study examined the repeated bout effect of two resistance training bouts on cycling efficiency and performance.

**Methods:**

Ten male resistance-untrained cyclists (age 38 ± 13 years; height 180.4 ± 7.0 cm; weight 80.1 ± 10.1; kg; *V*O_2max_ 51.0 ± 7.6 ml.kg^−1^.min^−1^) undertook two resistance training bouts at six-repetition maximum. Blood creatine kinase (CK), delayed-onset of muscle soreness (DOMS), counter-movement jump (CMJ), squat jump (SJ), submaximal cycling and time-trial performance were examined prior to (Tbase), 24 (T24) and 48 (T48) h post each resistance training bout.

**Results:**

There were significantly lower values for DOMS (*p* = 0.027) after Bout 2 than Bout 1. No differences were found between bouts for CK, CMJ, SJ and submaximal cycling performance. However, jump height (CMJ and SJ) submaximal cycling measures (ventilation and perceived exertion) were impaired at T24 and T48 compared to Tbase (*p* < 0.05). Net efficiency during submaximal cycling improved at Bout 2 (23.8 ± 1.2) than Bout 1 (24.3 ± 1.0%). There were no changes in cycling time-trial performance, although segmental differences in cadence were observed between bouts and time (i.e. Tbase vs T24 vs T48; *p* < 0.05).

**Conclusion:**

Cyclists improved their cycling efficiency from Bout 1 to Bout 2 possibly due to the repeated bout effect. However, cyclists maintained their cycling completion times during exercise-induced muscle damage (EIMD) in both resistance training bouts, possibly by altering their cycling strategies. Thus, cyclists should consider EIMD symptomatology after resistance training bouts, particularly for cycling-specific technical sessions, regardless of the repeated bout effect.

## Introduction

Cycling is a popular endurance sport that requires a range of physiological characteristics, including high oxygen uptake, muscular endurance, and muscular efficiency (Sanders and Heijboer 2019). Whilst cyclists primarily focus on endurance training to optimise performance, there is a growing body of evidence indicating that resistance training embedded within a cycling-specific training programme improves cycling time-trial performance (Aagaard et al. 2011). It is understood that resistance training enhances muscular strength, muscle fibre composition and neural unit recruitment, ultimately optimising cycling efficiency (Aagaard et al. 2011). However, resistance training may also result in exercise-induced muscle damage (EIMD), particularly if cyclists are unfamiliar with such a mode of exercise (Proske and Morgan 2001).

The eccentric contractions of resistance training cause the greatest levels of EIMD, by placing a high level of stress on the muscular tissue (Chen et al. 2011). The common symptoms of EIMD include increased creatine kinase (CK), stiffness and swelling, delayed-onset muscle soreness (DOMS) and decreased muscular contractility, which can last for several days (Clarkson et al. 1987). Although EIMD is known to impair a range of physical performance measures, studies have also shown reductions in cycling peak power output, with a concomitant increase in oxygen cost and ventilation for up to 48 h following muscle-damaging exercises (Burt and Twist 2011; Davies et al. 2009; Gleeson et al. 1995; Yunoki et al. 2011; Baranauskiene et al. 2017; Black and Dobson 2012). Therefore, cyclists may be unable to complete their cycling-specific training sessions at the appropriate intensity or duration during periods of EIMD, compromising training quality. Indeed, Doma et al. (2017a) indicated the need for endurance athletes to minimise the impact of EIMD caused by resistance training during concurrent training to limit sub-optimal endurance adaptations, also known as resistance-training induced sub-optimisation (Doma et al. 2019a). However, the studies that examined the effect of EIMD on cycling performance incorporated muscle-damaging exercises with repeated jump exercises, or isokinetic eccentric contractions (Black and Dobson 2012; Burt and Twist 2011; Twist and Eston 2009). These types of exercises may not elicit adaptations usually observed from resistance training sessions consisting of various exercises (e.g. back squats, leg extension and leg curls) with heavy external loads. Furthermore, the symptoms of EIMD are often reduced following the second bout of identical muscle-damaging exercises, referred to as the repeated bout effect (RBE) (Clarkson et al. 1987). Determining the impact of RBE using traditional resistance exercises may be more practical during concurrent training situations, especially for cyclists who are commencing, or are restarting resistance training from a training hiatus due to off-season or injury.

To date, the impact of RBE on cycling performance is yet to be determined as far as we are aware. However, several studies have reported that several multimodal resistance training bout (i.e. back squats, leg extension and leg curls) reduced the level of CK, DOMS and attenuation of muscular performance (Doma et al. 2015), whilst improved running economy and running time-to-exhaustion (Doma et al. 2017b). These findings suggest that adaptations to several multimodal resistance training bout improve running economy and performance, possibly due to the RBE, and are applicable for runners commencing a resistance training programme. However, these findings may not be translatable to cyclists as the physiological responses are clearly distinct between running and cycling (Thomas et al. 1995). Subsequently, this study examined the impact of traditional resistance training on cycling efficiency and performance across two bouts. It was hypothesised that cycling performance would be impaired to a greater level following the initial resistance training bout compared to the second resistance training bout due to the RBE. The practical implications of these findings will assist coaches and cyclists to modify resistance and cycling-specific training sessions, enhance training quality and optimise cycling performance.

## Methods

### Participants

Ten healthy men (age 38 ± 13 years; height 180.4 ± 7.0 cm; body mass 80.1 ± 10.1 kg; peak oxygen uptake 51.0 ± 7.6 mL·kg·min^−1^), all of whom were recreational or competitive cyclists (cycling between 150 and 300 km per week), volunteered to participate in the study. The cyclists were categorised as P3 as per the categorisation system by De Pauw et al. (2013). Cyclists that had participated in any lower body resistance training within the preceding six months were excluded. To control for biological variation, participants were required to wear the same shoes for all resistance training sessions and use the same bicycle and wear the same shoes for the cycling test sessions. Participants were also requested to refrain from ingesting caffeine within two hours of training and testing, avoid strenuous exercise for at least 24 h prior to training and testing, abstain from undertaking any recovery protocols during the entire study period (e.g. ice bath, compression, stretching, analgesic drugs, nutritional supplements) and maintain consistent sleep and nutrition habits throughout the study. All the training and assessments sessions were conducted at the same time of day, between the hours of 5:00 and 7:00am. The risks associated with the experimental procedures were explained to participants prior to involvement in the study and each participant completed a written informed consent and medical health questionnaire. The study was approved by the Institutional Human Research Ethics Committee and was conducted in accordance to the Declaration of Helsinki. Using a statistical package (G*Power 3.1.9.2, Heinrich-Heine-Universität, Düsseldorf, Germany), an a priori sample size calculation showed that at least 10 participants were deemed to be sufficient to detect a significant change in EIMD measures, including CK, DOMS and jump performance (Doma and Deakin 2013, 2014), and cycling time-trial performance (Burt and Twist 2011) with the power and alpha level set at 0.8 and 0.05, respectively.

### Research design

**Fig. 1:**
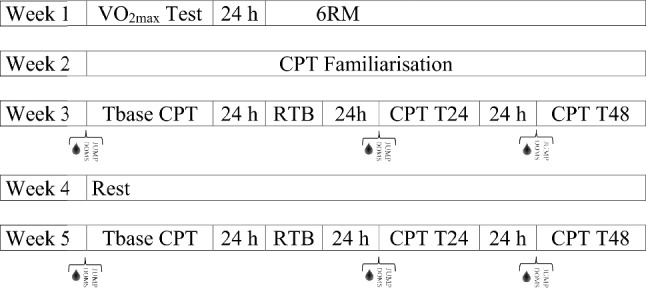
Schematic of the research design across 5 weeks with sessions separated by 24 h periods.  Blood collection;  Recording of delayed-onset of muscle soreness;  Countermovement jump, squat jump and drop jump

This study was conducted across five weeks, with a graded exercise test to assess maximal oxygen uptake test (*V*O_2max_) and a six-repetition maximum (6RM) assessment undertaken in the first week separated by at least one day (Fig. 1). The second week consisted of a familiarisation session to acquaint participants with the vertical jump assessments and cycling performance test. During the third week, the participants completed a resistance training bout, with CK, DOMS, jump performance test and cycling performance test sessions performed the day before the resistance training bout as baseline (Tbase), and again at 24 h (T24) and 48 h (T48) after the resistance training bout. After a minimum of two weeks of recovery, the participants repeated all protocols that were performed in the third week.

### Assessment of maximal oxygen uptake

Prior to the *V*O_2max_ test, a progressive warm-up was conducted using a commercial ergometer (KICKR, Wahoo, Atlanta, Georg., USA), with the participant’s personal bicycle attached. This ergometer has previously been reported with a high level of test–retest reliability and construct validity (Zadow et al. 2018). During the warm-up, the participants cycled at a self-selected pace for five minutes, followed by power increments of 15%, 30% and 45% of the self-selected power for one minute, respectively. The *V*O_2max_ test was conducted using a continuous, incremental method, starting at 100 W, and increased by 25W every minute until volitional exhaustion was reached using verbal encouragement. The *V*O_2max_ was defined as been reached when two of the following criteria were met: (a) respiratory exchange ratio (RER) ≥ 1.1; (b) peak heart rate (HR) within 10 b/min-1 of age predicted maximum; or (c) peak rating of perceived exertion (RPE) ≥ 19 (Doma et al. 2015). During the *V*O_2max_ test, expired air was collected using an indirect calorimetry system (Quark CEPT, Cosmed, Rome, Italy) to determine the second ventilatory threshold (VT_2_). The Cosmed system was calibrated using certified alpha gas mixtures of 16% oxygen and 4% carbon dioxide concentration and a 3 L calibration syringe. The VT_2_ was quantified by ascertaining the inflexion point of ventilation (*V*_E_) with respect to carbon dioxide production (VCO_2_) on a scatter diagram. The corresponding power output at VT_2_ was then used to establish the exercise intensity during the cycling performance test.

### Cycling performance test

**Fig. 2:**
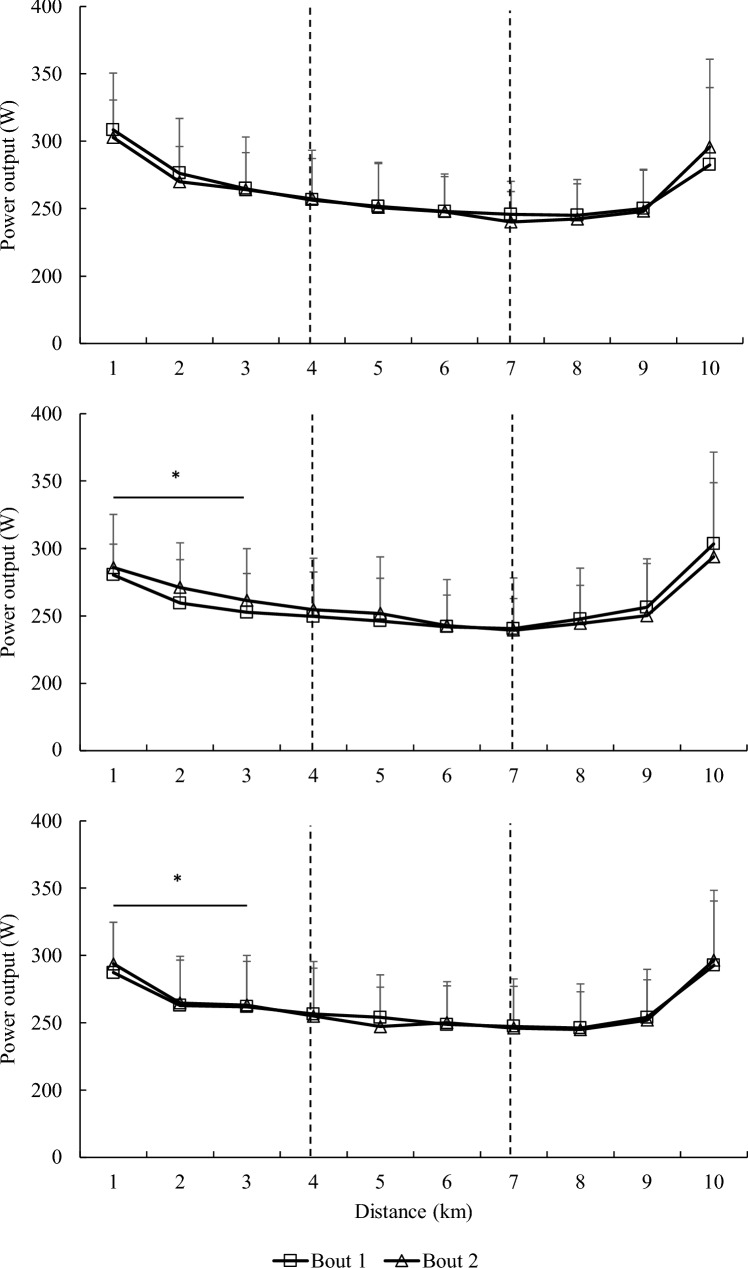
Average power output for each 1 km of the 10 km simulated time-trial at **a** Tbase, **b** T24 and **c** T48 h after Bout 1 and 2 of the traditional resistance training session. Values are means ± standard deviation. *Significantly lower than Tbase as a main effect of time

The same ergometer and bicycle in the *V*O_2max_ test were used for the cycling performance test protocol. Following a warm-up identical to that used in the *V*O_2max_, the cycling performance test protocol was conducted, consisting of two discontinuous, incremental stages cycling for 10 min at 70% and 90% of VT_2_*,* respectively, with 2 min of passive rest in-between each stage (Doma et al. 2012). The intensity at 70% replicated workload at long slow distance and 90% replicated the workload at pace tempo during typical cycling training sessions. Five minutes after the second stage at 90% of VT_2_, the participants performed a 10 km simulated time trial, using a flat course (Zwift Inc., Long Beach, USA) (Fig. 2). The measure of HR (Polar RS800, New York, USA) was collected and averaged during the last 5 min of the first and second stages of the cycling performance test, along with oxygen consumption (*V*O_2_) and *V*_E_ (Quark CEPT, Cosmed, Rome, Italy)*.* The measure of RPE was also collected in the final two minutes of each stage, whilst blood lactate concentration (LAC; Lactate Pro2, Arkray, Japan) was collected immediately following the 70 and 90% stages. During the time trial, time duration was collected along with HR (Polar RS800, New York, N). Average power and average cadence were collected and averaged for segments of 1–3 km, 4–6 km and 7–10 km, which allowed the time-trial to be analysed in further depth. The RPE values were then collected at 3 km, 6 km and 9 km of the time trial to align with the segments previously mentioned.

### Cycling efficiency

Data recorded from 70 and 90% of VT_2_ stages using an indirect calorimetry system (Quark CEPT, Cosmed, Rome, Italy) was used to determine gross efficiency (GE) and net efficiency (NE). Measures of *V*O_2_ and RER were averaged during the last 5 min of the stages to ensure participants reached steady-state cycling, which was defined as < 10% change in *V*O_2_ per minute. The GE and NE metrics were calculated from measures of energy expended, *V*O_2_ and work rate (Chavarren and Calbet 1999). The GE was calculated as the ratio of work accomplished.min^−1^ to energy expended.min^−1^, using the RER values from the tables of (Peronnet and Massicotte (1991) to determine the *V*O_2_ and energy equivalent for oxygen. The NE was calculated as the GE minus the energy expended when seated at rest on the bicycle (Matomaki et al. 2019). The GE was calculated as the mean of all data collected in the last 5 min of each stage. The ergometer maintained a fixed power output whilst the participants were asked to maintain a consistent cadence that was determined from the *V*O_2max_ test for all sessions and intensities.

### Indirect muscle damage markers

Delayed-onset muscle soreness (DOMS) was recorded with participants to determine their level of muscle soreness on a visual analogue scale from 1 to 10, displayed as “not sore” to “very sore” (Doma et al. 2015). The rating was scored as participants completed one body weight squat through full range (Doma et al. 2015). Blood creatine kinase (CK) activity was also measured as an indirect marker of muscle damage by collecting a 30 microL capillary blood sample via finger prick, pipetted immediately to a test strip and analysed with the use of a colorimetric assay procedure (Reflotron Plus, Mannheim, Germany). The intra-assay coefficient of variation for CK within our laboratory was determined as 7.2%. The participants also performed three vertical jump variants on a jump mat (Swift EZE Testing MatSwift Performance, Queensland, Australia) to determine countermovement jump (CMJ), a squat jump (SJ) from a seated position on a 30 cm box, and a drop jump (DJ) from a 30 cm box (Doma et al. 2017b). The participants completed three trials for each jump variant, with 30 s of rest interspersed between each trial and the best performance reported for analyses.

### Repetition maximum assessment

Prior to the 6RM test, participants completed a standardised warm-up on a cycle ergometer (Monark, 828E, Sweden) for 5 min at a comfortable pace followed by dynamic stretches of the lower extremity (i.e. leg swings in frontal and sagittal plane). Following the warmup, sequential 6RM tests were conducted for Smith-machine back squat (MPL 706, Maxim Fitness, Australia), single-leg leg press (NS4000, Nautilus), leg extension (NS4000, Nautilus) and leg curls (NS4000, Nautilus). Participants performed warm-up sets of 10 repetitions of each exercise at sub-maximal loads. Following the warm-up sets, participants completed 8–10 repetitions at near maximal workloads based on perceived effort using a 10-point visual analogue scale during the warm-up set. After 5 min of rest, loads were increased by 10–15% to attempt the 6RM test. The final load was achieved within 3–5 attempts with a 5 min rest period between each attempt. The range of motion was standardised for the squat and leg press exercises by recording displacement of the external load using markers, which were then noted and used in the resistance-training bout.

### Resistance training bout

During the resistance training bout, participants performed 3 sets of 6 repetitions in the order of Smith-machine back squat, single-leg press, leg extension and leg curls in a controlled manner using a typical resistance training approach of approximately 1 s of concentric and eccentric contractions. The resistance training intensity was set at 95% of 6RM to allow participants to complete each set without failure. A passive 2 min rest period was provided between each set and exercise. This resistance training protocol has been used previously across several studies (Doma et al. 2015, 2017b, 2019b).

### Statistical analyses

Data were reported as mean ± standard deviation and analysed using the statistical package of social sciences (SPSS, version 25; IBM Corp., Armonk, N.Y., USA). The Shapiro–Wilk test was conducted to test for normality, with the majority of data exhibiting normal distribution. Thus, a two-way (bout x time) repeated measures analysis of variance was used to compare indices of EIMD and cycling performance measures between time points (i.e. Tbase, 24 h and 48 h post-exercise) and the first and second resistance training bout. When an interaction effect, main effect of condition or main effect of time was identified, post hoc analyses were conducted using Bonferroni’s pairwise comparisons. Where main effects of bouts were identified, the corresponding means ± standard deviations were reported in the text in brackets. The alpha level was set at 0.05 for all inferential statistical analyses. To determine the test–retest reliability of the jump performance, cycling efficiency and cycling time-trial, intra-class correlation coefficient (2-way mixed effects model), was conducted by comparing measures between the two baseline time points. The ICC values greater than 0.9, ranging between 0.8 and 0.89 and under 0.80 were classified as excellent, moderate and questionable, respectively (Vincent 2005). The two baseline time points were also used to calculate standard error of measurement and minimal detectable change for absolute reliability.

## Results

### Reliability

The test–retest reliability and the absolute reliability are presented in Table 1. Overall, moderate to excellent test-reliability was evident for the jump performance measures (ICC of 0.88–0.97) and for most parameters of the cycling efficiency test (0.81–0.99), except for net efficiency at 70% of VT_2_ with questionable test–retest reliability (ICC of 0.79). The time during time trial performance exhibited excellent test–retest reliability (ICC of 0.99), whilst most of the parameters during the cycling time-trial demonstrated moderate to excellent test–retest reliability (ICC of 0.83–0.92), except for cadence at 4–6 km and 7–10 km with questionable test–retest reliability (ICC of 0.80 and 0.65, respectively).

**Table 1:** The test–retest reliability and absolute reliability of jump performance and cycling performance measures

	ICC	SEM	MDC
CMJ	0.94	1.62	4.46
DJ	0.97	0.68	1.84
SJ	0.88	0.42	1.16
*V*O_2_-70%	0.99	0.25	0.70
*V*O_2_-90%	0.89	0.39	1.09
VE-70%	0.98	0.69	1.92
VE-90%	0.96	1.73	4.80
BLA-70%	0.91	0.10	0.28
BLA-90%	0.96	0.32	0.87
RPE-70%	0.88	0.27	0.77
RPE-90%	0.88	0.24	0.65
HR-70%	0.86	0.92	2.56
HR-90%	0.84	0.92	2.55
GE-70%	0.83	0.21	0.59
GE-90%	0.88	0.19	0.53
NE-70%	0.79	0.90	2.50
NE-90%	0.81	0.79	2.18
TT	0.99	18.9	52.35
CAD 1–3 km	0.92	2.78	7.70
CAD 4–6 km	0.80	3.73	10.33
CAD 7–10 km	0.65	4.21	11.68
POW 1–3 km	0.88	16.38	45.39
POW 4–6 km	0.90	14.19	39.32
POW 7–10 km	0.83	34.38	95.29

*CMJ* countermovement jump, *DJ* drop jump, *SJ* squat jump, *VO*_2_*-70%* oxygen consumption at 70% of second ventilatory threshold (VT_2_), *VO*_2_*-90%* oxygen consumption at 90% of VT_2_, *VE-70%* ventilation at 70% of VT_2_, *VE-90%* ventilation at 90% of VT_2_, *BLA-70%* blood lactate at 70% of VT_2_, *BLA-90%* blood lactate at 90% of VT_2_, *RPE-70%* rating of perceived exertion at 70% of VT_2_, *RPE-90%* rating of perceived exertion at 90% of VT_2_, *HR-70%* heart rate at 70% of VT_2_, *HR-90%* heart rate at 90% of VT_2_, *GE-70%* gross efficiency at 70% of VT_2_, *GE-90%* gross efficiency at 90% of VT_2_, *NE-70%* net efficiency at 70% of VT_2_, *NE-90%* net efficiency at 90% of VT_2_, *TT* time trial, *CAD 1–3 km* cadence at 1–3 km during cycling time-trial, CAD 4–6 km cadence at 4–6 km during cycling time-trial, *CAD 7–10 km* cadence at 7–10 km during cycling time-trial, *POW 1–3 km* power output at 1–3 km during cycling time-trial, *POW 4–6 km* power output at 4–6 km during cycling time-trial, *POW 7–10 km* power output at 7–10 km during cycling time-trial

### Indirect muscle damage markers

For indirect markers of muscle damage, an interaction effect was identified for DOMS (*p* = 0.027), with significantly lower values during Bout 2 at T24 (*p* = 0.006) and T48 (*p* = 0.010) when compared to Bout 1 (Table 2). Furthermore, DOMS was significantly increased at T24 and T48 in Bout 1 (*p* < 0.01) and Bout 2 (*p* < 0.01) when compared to Tbase. No significant interaction effects were found for CK (*p* = 0.215), CMJ (*p* = 0.278), SJ (*p* = 0.825) or DJ (*p* = 898). However, there was a main effect of time for CMJ (*p* = 0.02; *η*_p_^2^ = 0.42) with significantly lower jump heights at T24 (25.5 ± 5.5 cm; *p* = 0.01) when compared to Tbase (28.3 ± 4.7 cm), although no significant differences were found at T48 (25.1 ± 5.7 cm; *p* = 0.12). A main effect of time was also found for SJ (*p* < 0.001; *η*_p_^2^ = 0.65), with significantly lower jump heights at T24 (25.5 ± 6.6 cm; *p* = 0.001) and T48 (25.5 ± 6.5 cm; *p* = 0.007) when compared to Tbase (28.0 ± 5.7 cm). There was no main effect of time for CK (*p* = 0.064; *η*_p_^2^ = 0.31) and DJ (*p* = 0.052; *η*_p_^2^ = 0.35), and no main effect of bout was identified for CK (*p* = 0.16; *η*_p_^2^ = 0.21), CMJ (*p* = 0.09; *η*_p_^2^ = 0.29), SJ (*p* = 0.38; *η*_p_^2^ = 0.09) or DJ (*p* = 0.14; *η*_p_^2^ = 0.23; Table 1).

**Table 2:** Changes in indirect markers of muscle damage

Parameter	Bout	Tbase	T24	T48	Bout × time interaction	Main time effect	Main bout effect
CK$\left( {U \cdot L^{ - 1} } \right)$	1	119.7 ± 46.7	417.0 ± 293.3	396.3 ± 606.2	*p* = 0.215	*p* = 0.06	*p* = 0.16
	2	133.3 ± 53.5	240.3 ± 128.9	150.2 ± 118.5	*η*_p_^2^ = 0.17	*η*_p_^2^ = 0.31	*η*_p_^2^ = 0.21
DOMS	1	1.4 ± 0.7	6.4 ± 2.0^a^	6.3 ± 2.4^a^	*p* = 0.027	*p* < 0.001	*p* = 0.001
	2	1.3 ± 0.5	4.8 ± 1.2^a,b^	4.5 ± 1.8^a,b^	*η*_p_^2^ = 0.330	*η*_p_^2^ = 0.81	*η*_p_^2^ = 0.70
CMJ (cm)	1	28.3 ± 5.0	24.6 ± 5.9^c^	24.5 ± 5.7	*p* = 0.278	*p* = 0.03	*p* = 0.086
	2	28.2 ± 4.3	26.4 ± 5.6	25.9 ± 6.0	*η*_p_^2^ = 0.13	*η*_p_^2^ = 0.42	*η*_p_^2^ = 0.29
SJ (cm)	1	27.7 ± 5.5	25.5 ± 7.4^c^	25.5 ± 6.6^c^	*p* = 0.825	*p* < 0.001	*p* = 0.38
	2	28.3 ± 6.2	25.5 ± 5.9	25.5 ± 6.7	*η*_p_^2^ = 0.02	*η*_p_^2^ = 0.65	*η*_p_^2^ = 0.09
DJ (cm)	1	28.6 ± 5.0	26.7 ± 5.4	25.9 ± 5.3	*p* = 0.898	*p* = 0.052	*p* = 0.14
	2	27.7 ± 3.9	25.7 ± 4.8	25.2 ± 4.4	*η*_p_^2^ = 0.004	*η*_p_^2^ = 0.35	*η*_p_^2^ = 0.23

*CK* blood creatine kinase, *DOMS* delayed onset of muscle soreness, *CMJ* countermovement jump, *SJ* squat jump, *DJ* drop jump, *Tbase* baseline, *T24* 24 h after the resistance training bout, *T48* 48 h after the resistance training bout

*η*_p_^2^ = eta partial squared

^a^Significantly different from Tbase (*p* < 0.05)

^b^Significantly different from Bout 1 (*p* < 0.05)

^c^Significantly different from Tbase as a main effect of time (*p* < 0.05)

### Sub-maximal cycling performance

No bout x time interaction effect was found for any cycling performance test measures (*p* > 0.05; Tables 3 and 4). However, there was a main effect of time for *V*_E_ at 70% VT_2_, with significantly higher values at T24 (74.48 ± 10.45 L.min^−1^) and T48 (73.76 ± 9.4 L.min^−1^) compared to Tbase (72.01 ± 10.20 L.min^−1^) and RPE during the 90% stage with T24 (16.40 ± 1.95) and T48 (16.45 ± 1.45) being significantly higher than Tbase (15.25 ± 1.54; *p* < 0.05). However, a main effect of time was not identified for all other variables during submaximal cycling (*p* > 0.05). There was a main effect of bout for NE, with a significant increase from Bout 1 to Bout 2 during the 90% stage (23.8 ± 1.2 vs 24.3 ± 1.0%; *p* < 0.05; Table 3) but no difference for GE and NE at any other stages.

**Table 3:** Changes in physiological, metabolic, and perceptual responses during cycling intensities corresponding to 70% of the second ventilatory threshold

Parameter	Bout	Tbase	T24	T48	Bout × time interaction	Main time effect	Main bout effect
*V*O_2_ (ml kg^−1^ min^−1^)	1	34.86 ± 5.47	35.58 ± 6.21	35.23 ± 5.83	*p* = 0.400	*p* = 0.23	*p* = 0.25
	2	34.92 ± 6.08	35.13 ± 5.65	34.65 ± 5.95	*η*_p_^2^ = 0.10	*η*_p_^2^ = 0.15	*η*_p_^2^ = 0.14
*V*_E_ (l min^−1^)	1	72.1 ± 9.8	74.7 ± 10.7^c^	73.8 ± 9.2^c^	*p* = 0.961	*p* = 0.04	*p* = 0.77
	2	71.9 ± 10.9	74.3 ± 10.5	73.8 ± 10.1	*η*_p_^2^ = 0.004	*η*_p_^2^ = 0.30	*η*_p_^2^ = 0.01
HR (beats min^−1^)	1	133.5 ± 6.1	136.9 ± 6.8	133.9 ± 6.8	*p* = 0.553	*p* = 0.051	*p* = 1.00
	2	134.5 ± 5.8	135.7 ± 6.4	134.1 ± 5.5	*η*_p_^2^ = 0.06	*η*_p_^2^ = 0.28	*η*_p_^2^ = 0.00
BL (mmol l^−1^)	1	2.0 ± 0.8	2.0 ± 0.7	1.8 ± 0.5	*p* = 0.397	*p* = 0.87	*p* = 0.71
	2	2.0 ± 0.7	1.9 ± 0.6	2.0 ± 0.8	*η*_p_^2^ = 0.10	*η*_p_^2^ = 0.02	*η*_p_^2^ = 0.02
RPE	1	11.5 ± 2.2	12.9 ± 2.3	12.9 ± 1.3	*p* = 0.241	*p* = 0.08	*p* = 1.00
	2	12.0 ± 1.2	12.6 ± 1.7	12.7 ± 1.3	*η*_p_^2^ = 0.15	*η*_p_^2^ = 0.29	*η*_p_^2^ = 0.00
GE (%)	1	20.6 ± 1.7	20.1 ± 1.4	20.3 ± 1.1	*p* = 0.630	*p* = 0.21	*p* = 0.93
	2	20.4 ± 0.7	20.2 ± 1.1	20.4 ± 0.6	*η*_p_^2^ = 0.05	*η*_p_^2^ = 0.16	*η*_p_^2^ = 0.001
NE (%)	1	24.3 ± 1.9	23.9 ± 1.7	24.3 ± 1.2	*p* = 0.904	*p* = 0.36	*p* = 0.63
	2	24.4 ± 1.1	24.1 ± 1.2	24.4 ± 0.6	*η*_p_^2^ = 0.01	*η*_p_^2^ = 0.11	*η*_p_^2^ = 0.03

*VO*_2_ oxygen consumption, *V*_*E*_ ventilation, *BL* blood lactate, *RPE* rating of perceived exertion, *GE* gross efficiency, *NE* net efficiency, *Tbase* baseline, *T24* 24 h after the resistance training bout, *T48* 48 h after the resistance training bout

*η*_p_^2^ = eta partial square

^c^Significantly different from Tbase as a main effect of time (*p* < 0.05)

**Table 4:** Changes in physiological, metabolic, and perceptual responses during cycling intensities corresponding to 90% of the second ventilatory threshold

Parameter	Bout	Tbase	T24	T48	Bout × time interaction	Main time effect	Main bout effect
*V*O_2_ (ml kg^−1^ min^−1^)	1	43.29 ± 6.80	43.54 ± 7.26	43.89 ± 7.31	*p* = 0.570	*p* = 0.71	*p* = 0.17
	2	42.89 ± 6.99	43.12 ± 6.93	42.86 ± 6.75	*η*_p_^2^ = 0.05	*η*_p_^2^ = 0.04	*η*_p_^2^ = 0.20
*V*_E_ (l min^−1^)	1	108.6 ± 22.6	113.6 ± 27.6	108.6 ± 22.1	*p* = 0.780	*p* = 0.10	*p* = 0.44
	2	106.9 ± 19.6	111.1 ± 22.6	108.4 ± 20.1	*η*_p_^2^ = 0.03	*η*_p_^2^ = 0.23	*η*_p_^2^ = 0.07
HR (beats min^−1^)	1	156.9 ± 5.6	159.8 ± 7.0	157.0 ± 6.3	*p* = 0.759	*p* = 0.06	*p* = 0.08
	2	157.3 ± 5.9	158.7 ± 6.8	156.8 ± 3.2	*η*_p_^2^ = 0.03	*η*_p_^2^ = 0.27	*η*_p_^2^ = 0.008
BL (mmol l^−1^)	1	6.1 ± 4.5	6.9 ± 5.4	5.3 ± 3.8	*p* = 0.110	*p* = 0.07	*p* = 0.12
	2	5.8 ± 3.6	5.8 ± 4.6	4.8 ± 2.9	*η*_p_^2^ = 0.22	*η*_p_^2^ = 0.30	*η*_p_^2^ = 0.25
RPE	1	15.2 ± 1.8	17.0 ± 2.0^a^	16.6 ± 2.0^a^	*p* = 0.121	*p* = 0.002	*p* = 0.09
	2	15.3 ± 1.6	15.9 ± 2.1	16.4 ± 1.3	*η*_p_^2^ = 0.21	*η*_p_^2^ = 0.51	*η*_p_^2^ = 0.29
GE (%)	1	20.9 ± 1.1	20.8 ± 1.3	20.7 ± 1.1	*p* = 0.441	*p* = 0.30	*p* = 0.07
	2	21.4 ± 1.1	20.9 ± 1.1	21.1 ± 0.9	*η*_p_^2^ = 0.08	*η*_p_^2^ = 0.13	*η*_p_^2^ = 0.33
NE (%)	1	23.8 ± 1.3^b^	23.8 ± 1.5	23.7 ± 1.2	*p* = 0.429	*p* = 0.56	*p* = 0.03
	2	24.6 ± 1.5	24.1 ± 1.1	24.2 ± 1.0	*η*_p_^2^ = 0.09	*η*_p_^2^ = 0.06	*η*_p_^2^ = 0.43

*VO*_2_ oxygen consumption, *V*_*E*_ ventilation, *BL* blood lactate, *RPE* rating of perceived exertion, *GE* gross efficiency, *NE* net efficiency, *Tbase* baseline, *T24* 24 h after the resistance training bout, *T48* 48 h after the resistance training bout

*η*_p_^2^ = eta partial square

^a^Significantly different from Tbase as a main effect of time (*p* < 0.05)

^b^Significantly different from Bout 2 as a main effect of bout (*p* < 0.05)

### Cycling time-trial performance

**Fig. 3:**
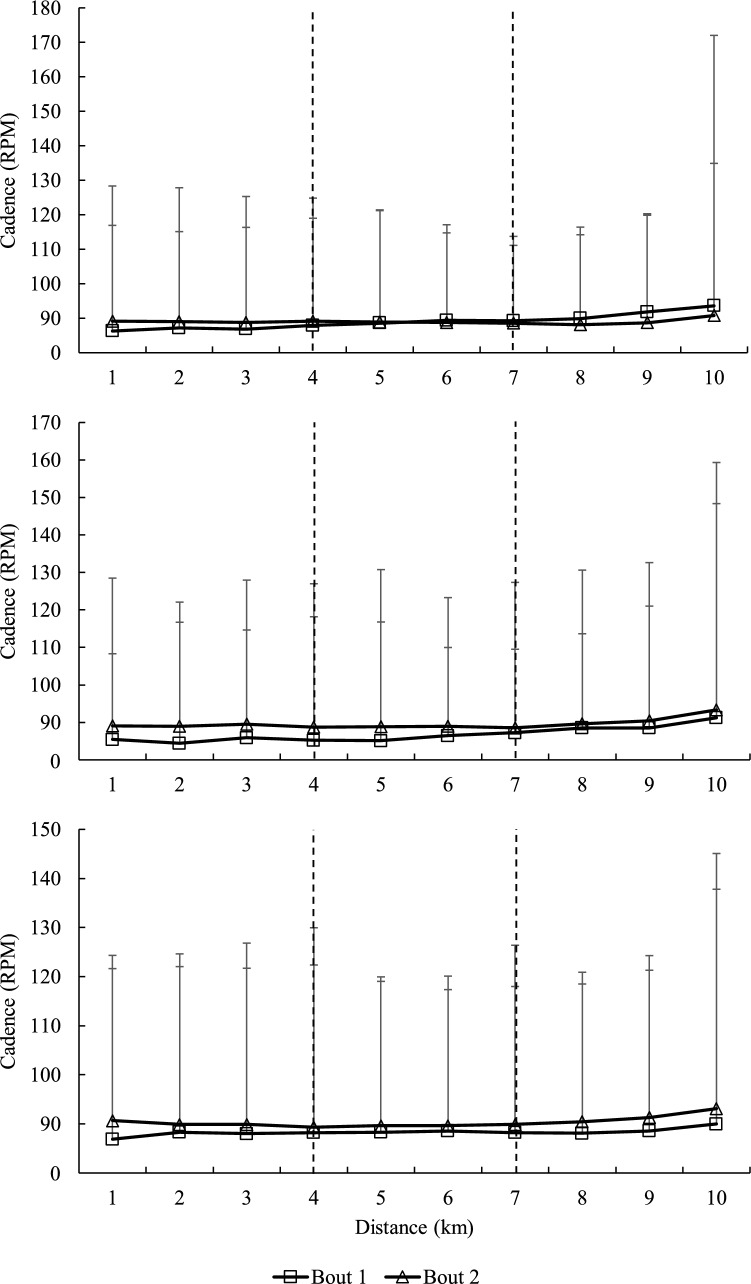
Average cadence for each 1 km of the 10 km simulated time-trial at **A** Tbase, **B** T24 and **C** T48 h after Bout 1 and 2 of the traditional resistance training session. Values are means ± standard deviation

Duration, average power output, HR and RPE are presented in Table 5. Average power output and cadence at each 1 km of the 10 km simulated time-trial, reported as 1–3 km, 4–6 km and 7–10 km segments, are displayed in Figs. 2 and 3. There was neither interaction effect, main effect of time nor main effect of bout for time-trial duration, average power output, HR and RPE (*p* > 0.05; Table 4). There were no interaction effects for average power output at the 1–3 km (*p* = 0.57; *η*_p_^2^ = 0.07), 4–6 km (*p* = 0.58; *η*_p_^2^ = 0.07) and 7–10 km (*p* = 0.53; *η*_p_^2^ = 0.08) segments (Fig. 2). Average power for 1–3 km showed a main effect of time with a significant decrease at T24 (268.5 ± 30.13 W;* p* < 0.05) and T48 (272.2 ± 31.28 W;* p* < 0.05) compared to baseline (281.1 ± 34 W). However, average power at 4–6 km and 7–10 km segments showed no significant differences between baseline, T24 and T48 as a main effect of time (*p* > 0.05), nor was there a main effect of bout at any segments for average power (*p* > 0.05). For average cadence, there were no interaction effects at the 1–3 km (*p* = 0.81; *η*_p_^2^ = 0.01), 4–6 km (*p* = 0.57; *η*_p_^2^ = 0.07) and 7–10 km (*p* = 0.24; η_p_^2^ = 0.16) segments (Fig. 3). There was a main effect of bout for average cadence at the 1–3 km segment (*p* = 0.01; *η*_p_^2^ = 0.55), with a significant increase from Bout 1 to 2 (86.7 ± 6.0 vs 89.4 ± 6.85 RPM). However, there were no main effects of time nor bout (*p* > 0.05) for average cadence at the 4–6 km and 7–10 km segments.

**Table 5:** Changes in time-trial cycling performance

Parameter	Bout	Tbase	T24	T48	Bout × time interaction	Main time effect	Main bout effect
Duration (s)	1	934 ± 33	941 ± 39	934 ± 39	*p* = 0.884	*p* = 0.50	*p* = 0.21
	2	944 ± 34	949 ± 51	942 ± 36	*η*_p_^2^ = 0.01	*η*_p_^2^ = 0.07	*η*_p_^2^ = 0.17
Avg power output (W)	1	259 ± 35	250 ± 35	254 ± 37	*p* = 0.834	*p* = 0.21	*p* = 0.77
	2	254 ± 36	251 ± 42	254 ± 38	*η*_p_^2^ = 0.02	*η*_p_^2^ = 0.16	*η*_p_^2^ = 0.01
HR (beats min^−1^)	1	163.4 ± 7.6	165.4 ± 7.1	165.0 ± 7.0	*p* = 0.826	*p* = 0.19	*p* = 0.65
	2	164.4 ± 8.0	165.9 ± 8.0	164.7 ± 5.9	*η*_p_^2^ = 0.02	*η*_p_^2^ = 0.17	*η*_p_^2^ = 0.02
RPE 3 km	1	15.9 ± 2.3	16.7 ± 2.2	16.6 ± 1.8	*p* = 0.502	*p* = 0.04	*p* = 0.40
	2	16.5 ± 1.6	16.7 ± 1.6	16.8 ± 1.5	*η*_p_^2^ = 0.07	*η*_p_^2^ = 0.29	*η*_p_^2^ = 0.08
RPE 6 km	1	17.2 ± 1.6	17.6 ± 1.8	17.8 ± 1.3	*p* = 0.831	*p* = 0.07	*p* = 0.19
	2	17.7 ± 1.3	17.7 ± 1.8	18.2 ± 1.1	*η*_p_^2^ = 0.02	*η*_p_^2^ = 0.25	*η*_p_^2^ = 0.18
RPE 9 km	1	18.5 ± 1.3	18.9 ± 1.4	18.9 ± 1.3	*p* = 0.539	*p* = 0.21	*p* = 0.31
	2	18.8 ± 1.3	18.7 ± 1.7	19.3 ± 0.7	*η*_p_^2^ = 0.07	*η*_p_^2^ = 0.16	*η*_p_^2^ = 0.11

*HR* heart rate, *RPE* rating of perceived exertion, *Avg* average, *Tbase* baseline, *T24* 24 h after the resistance training bout, *T48* 48 h after the resistance training bout

*η*_p_^2^ = eta partial square

## Discussion

This study examined the RBE of commonly practised resistance training on cycling efficiency and performance. The findings showed that the resistance training bout caused a significant increase in DOMS along with a reduction in CMJ and SJ height. However, limited differences were observed for sub-maximal cycling performance, with increases only observed in ventilation and RPE. Whilst the initial resistance training bout increased selected measures (*V*_E_ and RPE) during sub-maximal cycling at 24 h post-exercise, comparable increases were seen following the second bout, suggesting that these measures increased during sub-maximal cycling for 24 h post-exercise although no differences were found between the resistance training bouts. However, NE was increased following the second bout, indicating that cycling efficiency improved with a concomitant reduction in the level of EIMD, possibly due to the RBE phenomenon. Time-trial performance showed no changes following the resistance training bouts, nor between resistance training bouts. However, the average cadence increased during the second bout between the 1–3 km segment, with the cyclists able to pedal at a higher cadence to achieve the same time-trial duration.

Following the completion of a resistance training bout, changes in several indirect markers of muscle damage were observed for up to 48 h post-exercise, with a significant increase in DOMS and reduction in CMJ and SJ height. Whilst changes in CMJ and SJ between bouts were identified as main effects, the test–retest reliability between the two baseline time points were excellent, suggesting that changes between the bouts likely occurred after the resistance training bouts at T24 and T48. These findings are comparable to recent studies observing muscle damage and changes in muscular function for several time points following commonly practised resistance training bouts consisting of various lower body exercises (Doma et al. 2019b, 2021). The elevated measure DOMS suggests that the participants experienced EIMD following resistance training bouts, which may have compromised their jumping capacity possibly due to impaired muscular contractility (Twist and Eston 2007). Interestingly, there were no differences in DJ in the current study, neither between bouts nor across time points within bouts. These results conflict with a recent study (Harrison et al. 2022) reporting impaired DJ performance 24 h after the initial resistance training bout consisting of similar exercises to the current study (i.e. various lower extremity resistance exercises). Furthermore, Harrison et al. (2022) showed DJ improved after the second resistance training bout, supporting the RBE trend. The DJ protocol requires reactive strength attributes by optimising stretch–shortening cycle mechanics, when compared to CMJ and SJ (Ref). As such, the discrepancy in findings between the study by Harrison et al. (2022) and the current study may be due to distinct training backgrounds of the participants. In the study by Harrison et al. (2022), the participants were fast bowlers in cricket, that predominantly necessitates anaerobic power by optimising stretch–shortening cycle mechanics (Ramachandran et al. 2021). Conversely, our participants were cyclists with a stronger endurance training background, that requires lower anaerobic demands.

For the sub-maximal cycling performance measures, there was a significant increase in *V*_E_ along with an increase after the resistance training bouts. Previous research has suggested the link between ventilatory response and DOMS and suggested that discharge of group III and IV afferent fibres near blood vessels in the muscle increases ventilation (Burt and Twist 2011). Thus, the increased ventilation following the resistance training bouts reported in this study may be a result of an increase in the level of DOMS. Overall, the findings from the current study align with a recent meta-analysis (Devantier-Thomas et al. 2023), with an increase in *V*_E_ and RPE during submaximal cycling performed during EIMD. However, it is important to note that we did not conduct correlations between *V*_E_ and RPE, due to lack of sample size and such analysis was beyond the scope of this study. Nonetheless, further research is warranted to confirm the linkage between *V*_E_ and RPE changes in response EIMD. Interestingly, there were no changes in the other physiological measures (*V*O_2_ and HR) in our findings, which differs to those of previous studies where an increase in physiological cost of sub-maximal cycling was reported during periods of EIMD, including *V*O_2_ and HR (Burt and Twist 2011; Davies et al. 2009; Gleeson et al. 1995; Yunoki et al. 2011; Baranauskiene et al. 2017; Black and Dobson 2012). The discrepancy in findings may be attributed to the differences in the volume of work involved in the muscle-damaging protocols. For example, Baranauskiene et al. (2017) used a muscle-damaging protocol consisting of 100 drop jumps, which has a greater level of metabolic demand due to a greater number of rapid eccentric contractions than the current study. Furthermore, Burt and Twist (2011) included 10 sets of 10 repetitions of back squats, totalling 100 repetitions, which is substantially greater than the total number of repetitions in our resistance training bout with 72 repetitions. Therefore, their muscle-damaging protocols may have a larger impact on *V*O_2_ responses during periods of EIMD.

An important component of this study was the inclusion of a 10 km simulated time-trial, given the limited research on the effects of EIMD on cycling time-trial performance. According to the current findings, the resistance training bout caused no changes in time-trial performance, which supports previous studies (Karasiak and Guglielmo 2018; Silva-Cavalcante et al. 2019), reporting no differences in 5 min distance-trial and 4 km time-trial protocols, respectively, during periods of EIMD amongst cyclists. Interestingly, studies that involved non-cycling populations reported a decrease in time-trial performance during periods of EIMD (Burt and Twist 2011; Twist and Eston 2009). As such, it appears that the changes in cycling time-trial performance in response to EIMD protocols may be more apparent in individuals with a lesser cycling background. As such, participants with cycling backgrounds may have a greater ability to adapt to an adverse condition by altering their motor recruitment patterns as a compensatory mechanism to maintain time-trial performance. In fact, our findings further support this conjecture, with a significant increase in cadence during the second resistance training bout, compared to the first resistance training bout, during the 1–3 km segment. Furthermore, whilst the differences between bouts were based on main effects of bout, the excellent test–retest reliability reported between baseline time points with an ICC value of 0.92 for average cadence at the 1–3 km segment further confirmed that differences between bouts were likely after the resistance training bouts at T24 and T48. This suggests that cyclists manipulated the cadence to help maintain overall cycling performance. Previous research has also shown the ability of cyclists to adapt to pedalling cadence and technique to maintain overall performance (Lucia et al. 2001). The interplay between pedal force and cadence is the determinant of the overall power output. As pedal force is reducing, it is offset by an increase in cadence to maintain power output (Abbiss et al. 2009). The current study observed changes in cadence whilst maintaining power output between bouts, which suggests that pedal force may also be manipulated. Whilst not directly measured, it is possible that cyclists increased their average cadence to offset the reduction in force output and maintain power output from Bout 1 to Bout 2. These changes may not represent the conventional theory of the RBE trend, with increased force generation capacity typically observed after the second muscle-damaging bout due to a reduction in the level of EIMD and enhanced recovery kinetics (Doma et al. 2015). However, it is important to note that there were no changes in any of the jump performance measures between Bout 1 and Bout 2 in our study. Furthermore, the link between jump performance and the cycling time-trial test may not be straightforward, considering that a jump performance test requires one repetition of maximal effort, as opposed to a cycling time-trial test involving continuous submaximal contractions. The disparity between explosive strength measures and submaximal aerobic exercise protocols has in fact been reported previously, with changes in CMJ performance, despite any lack of changes in running economy measures for 24–48 h after muscle-damaging protocols (Doma et al. 2015). Therefore, the interplay between cadence and force output, which are the products of power output, appears to be complex, and it is difficult to assume with certainty that force output was reduced to the same magnitude as cadence in our study. Further research is required to determine the interaction between cycling cadence and force output during periods of EIMD. Nonetheless, we can deduce that the participants altered their cycling strategy from the first to the second bout due to changes in cadence, which may partly explain the ability of cyclists to maintain cycling time-trial performance despite the presence of EIMD, by manipulating cycling mechanics.

A novel component of this study was the inclusion of a second resistance training bout to examine the RBE. A reduction in DOMS following the second bout was observed (Table 1), along with an increase in NE during the 90% VT_2_ and increased cadence during the 1–3 km segment of the time trial. The increase in NE suggests that the cyclists may have improved their cycling efficiency following the second bout, possibly due to a reduction in the level of EIMD. These findings demonstrate the benefit of cyclists with minimal resistance training background to undertake multiple resistance training bouts to mitigate high level of EIMD and enhance cycling efficiency post-exercise. However, it is also essential to note that there were no significant differences in *V*_E_ and RPE between bouts, even though these measures increased following the resistance training bouts as a main effect. Furthermore, there is limited support of our findings from previous studies as our study is the first to investigate the RBE of resistance training on cycling performance. Nonetheless, if our results are compared to previous findings with other modes of endurance exercise, similar results have been reported. For example, the work by Doma et al. (2015) observed significant impairment in sub-maximal running performance following two resistance training bout’s consisting of similar exercises (incline back squats, leg press, leg extension and leg curls), although no differences were identified between bouts. However, when Doma et al. (2017b) incorporated a third resistance training bout in a follow-up study, sub-maximal running performance was improved, suggesting that at least three resistance training bouts were necessary to exhibit protective effects from EIMD on sub-maximal running performance, also referred to as the repeated-repeated bout effect. Thus, it is possible that, had the current study incorporated a third resistance training bout, a significant reduction in *V*_E_ and RPE, and possibly other physiological measures, may have been observed during sub-maximal cycling. However, further research is warranted to confirm this possibility.

It is necessary to acknowledge some of the limitations of the current study. First, the findings of the study have limited transferability to other endurance sports due to the differences in exercise modes (i.e. lower limb impact present during running). However, the current study provides findings specific to cycling performance which are missing in the current literature. Second, cycling performance tests were undertaken in a laboratory environment, and as such, may be different to the environment in which most cyclists train. However, the laboratory-setting also allows for a more controlled environment, by preventing the influence of external factors, such as ambient temperature, wind resistance and terrain. Furthermore, we ensured the participants were familiar with the environment, which included the use of a personal bicycle. Finally, the resistance training bout was prescribed using a six-repetition range that has been shown to exhibit EIMD symptomatology (Doma et al. 2015, 2017b, 2019b). Thus, the acute responses of resistance training on cycling performance reported in the current study may not be reproducible to resistance training bout set at other training intensity or volume, which warrants further research.

In conclusion, this study found that a traditional resistance training bout caused partial EIMD in cyclists with increased DOMS and decreased explosive strength at 24 and 48 h following the bouts. The NE during the sub-maximal cycling protocol improved after the second bout, suggesting an RBE trend. However, VE and RPE were equally perturbed following two resistance training bouts, with an increased number of sessions possibly required to induce a clearer RBE trend for sub-maximal cycling performance. Time-trial performance showed no changes, however cyclists manipulated cadence and power to achieve performance when in the presence of EIMD. Based on the current findings, when prescribing resistance training to trained cyclists for the first time, they should avoid sub-maximal cycling until after the second resistance training bout to enhance cycling efficiency. However, cycling sessions that require cyclists to perform at maximal effort, or when performing time-trial, should be avoided for 48 h post-exercise for at least the first two resistance training bouts. This is because the quality of cycling sessions may be reduced due to EIMD, particularly for sessions with a focus on the technical elements of cycling performance, such as pacing.

## Data Availability

Data will be made available upon request.
